# Thick Skin on the Dorsal Spine in Osteoproliferative Disease: Ossification of the Posterior Longitudinal Ligament and Diffuse Idiopathic Skeletal Hyperostosis

**DOI:** 10.7759/cureus.62235

**Published:** 2024-06-12

**Authors:** Tadatsugu Morimoto, Takaomi Kobayashi, Hirohito Hirata, Kazunari Sugita, Permsak Paholpak, Masatsugu Tsukamoto, Shun Umeki, Tomohito Yoshihara, Yu Toda

**Affiliations:** 1 Department of Orthopedic Surgery, Faculty of Medicine, Saga University, Saga, JPN; 2 Department of Orthopedics, Saga University, Saga, JPN; 3 Division of Dermatology, Department of Internal Medicine, Faculty of Medicine, Saga University, Saga, JPN; 4 Department of Orthopedics, Faculty of Medicine, Khon Kaen University, Khon Kaen, THA

**Keywords:** osteoporosis, diffuse idiopathic skeletal hyperostosis, ossification of the posterior longitudinal ligament, osteoproliferative disease, skin thickness

## Abstract

Background

Although the correlation between reduced skin thickness and reduced bone density has been investigated, no study has evaluated skin thickness and osteoproliferative diseases, including ossification of the posterior longitudinal ligament (OPLL) and diffuse idiopathic skeletal hyperostosis (DISH).

Methodology

This retrospective cohort study consisted of 99 consecutive patients aged ≥60 years treated for spinal surgery at our hospital between January 2022 and March 2023. Skin thickness was measured at the dorsal side of the cervical, thoracic, and lumbar vertebrae on the sagittal cross-section image of whole-spine CT. Based on the median value, skin thickness was categorized into two groups based on a median thickness of 4 mm. Bone mineral density (BMD) was assessed. The sum of the vertebral body and intervertebral bridging osteophytes of the anterior longitudinal and posterior longitudinal ligament were defined as the OALL index and OPLL index. Serum levels of bone metabolism-related markers, such as tartrate-resistant acid phosphatase type 5b, procollagen I N-propeptide, 25-hydroxyvitamin D, and periostin, were measured. To assess the association between skin thickness and imaging findings, we calculated the adjusted odds ratios, adjusting for age, sex, and body mass index (BMI) and using univariate and multivariate logistic regression analyses.

Results

No significant differences were found in skin thickness in the three dorsal regions of the cervical, thoracic, and lumbar spine (median = 3.3 mm versus 3.5 mm versus 3.4 mm, p = 0.357) and bone metabolism-related markers. Adjusting for age, sex, and BMI, cervical, thoracic, and lumbar skin thicknesses were related to DISH, the OPLL index, and the OPLL and OPLL index, respectively.

Conclusions

Skin thickness did not correlate with BMD but with the amount of spinal ossification. A correlation was found between skin thickness and vertebral and intervertebral ossification; vertebral osteophytes, OPLL, and DISH may be more common in thicker skin.

## Introduction

With the emergence of a super-aging society, degenerative bone metabolic diseases such as osteoporosis and osteoproliferative diseases, including ossification of the posterior longitudinal ligament (OPLL) and diffuse idiopathic skeletal hyperostosis (DISH), have increased in number and garnered more attention [1-3]. While factors such as genetics, aging, sex, endocrinology, metabolism, inflammation, and lifestyle have been implicated in these bone metabolism diseases [1-3], their underlying pathology remains elusive. Gaining insights into the complexities of these conditions is essential not only for relieving the burdens on patients, healthcare providers, and society but also for enabling early diagnosis and intervention.

The skin is continuous and soft on the body surface, whereas the bones are hard and separate into more than 200 forms in the body [4]. Despite these obvious differences in appearance and composition, both skin and bones exhibit remarkable similarities [4]. They share common structural features and cellular functions, influenced by a shared matrix.

The human skin, averaging a 2 mm thickness, comprises layers of the epidermis (0.05-1.5 mm) and dermis (0.3-3.0 mm), with keratinocytes accounting for 90% of the epidermis and fibroblasts being the primary cellular component of the dermis [5]. Similarly, in bone formation, osteoblasts and specialized fibroblasts play a pivotal role by secreting and calcifying a specific matrix [4]. Both the skin and bones are dynamic organs undergoing continuous metabolic processes. Fibroblasts and keratinocytes in the skin, as well as osteoblasts (a subtype of fibroblast) in the bones, utilize Wnt signaling to synthesize collagen, the principal organic constituent responsible for maintaining strength and protecting the body from external damage [4,6,7].

Skin symptoms often serve as indicators of various underlying medical conditions [8-10]. As early as 1919, it was observed that the skin acts as a mirror, reflecting fundamental truths about the pathogenesis of common diseases [8]. In other words, the skin serves as a window into internal bodily processes, often manifesting the initial signs or symptoms of systemic diseases, such as metabolic, cardiovascular, renal, gastrointestinal, and oncological disorders, which may be the sole indication of an underlying systemic condition [8-10]. Aurégan et al. [9] conducted a systematic review in 2018, revealing a moderate correlation between decreased skin thickness and reduced bone mineral density (BMD). While previous studies have primarily focused on the connection between skin thinning, BMD, and osteoporosis, the association between bone metabolism-related markers, collagen, and bone proliferative diseases such as OPLL and DISH has been underexplored. Therefore, this study aims to shed light on the relationship between skin thickness, BMD, bone turnover markers, and osteoproliferative diseases (OPLL and DISH).

## Materials and methods

Study design and patients

This retrospective cohort study included 159 consecutive patients aged 60 years or older who underwent spinal surgery at our hospital from January 2022 to March 2023. Patients with vertebral fractures (n = 18), atlantoaxial joint subluxation (n = 1), bone metastasis (n = 8), spinal tumors (n = 3), or insufficient evaluable data (n = 30) were excluded from this study. Ultimately, 99 patients with degenerative spinal diseases (cervical, 16; thoracic, 6; thoracolumbar, 2; lumbar, 75) were deemed eligible and their data were included in the analysis. This study received approval from the Saga University Clinical Research Review Board (approval number: #2023-06-R-01).

Skin thickness

**Figure 1:**
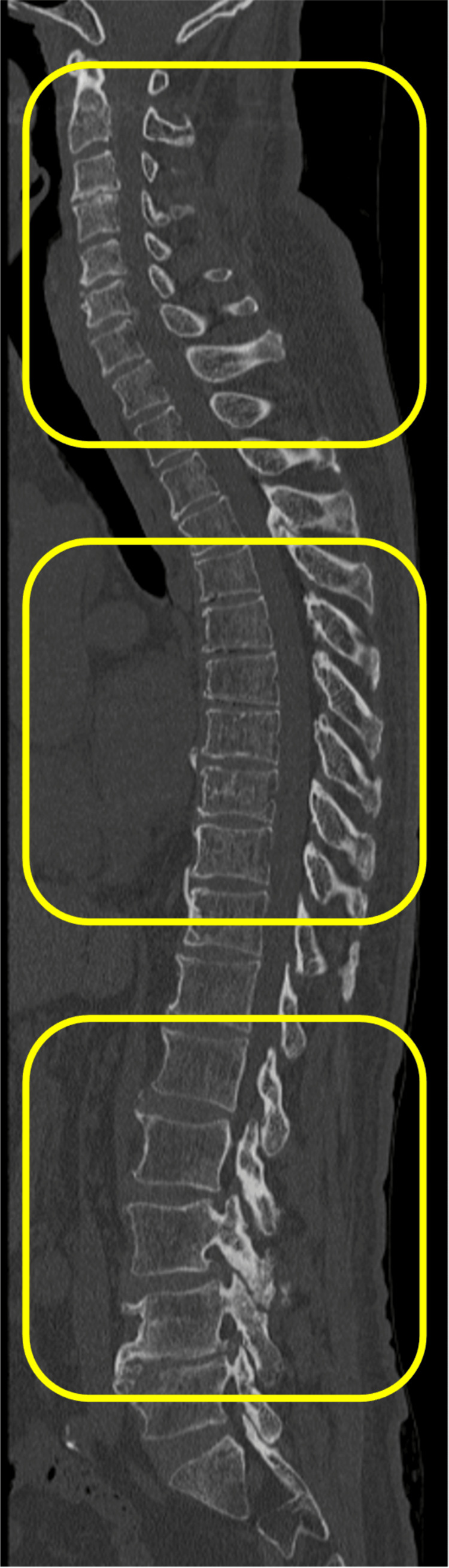
Measurement of cervical, thoracic, and lumbar spine skin thickness using sagittal whole-spine CT. The average skin thickness at the cervical (4-5, C5-6, C6-7 disc levels), thoracic (T7-8, T8-9, T9-10 disc levels), and lumbar (L2-3, L3-4, L4-5 disc levels) is defined as skin thickness.

**Figure 2:**
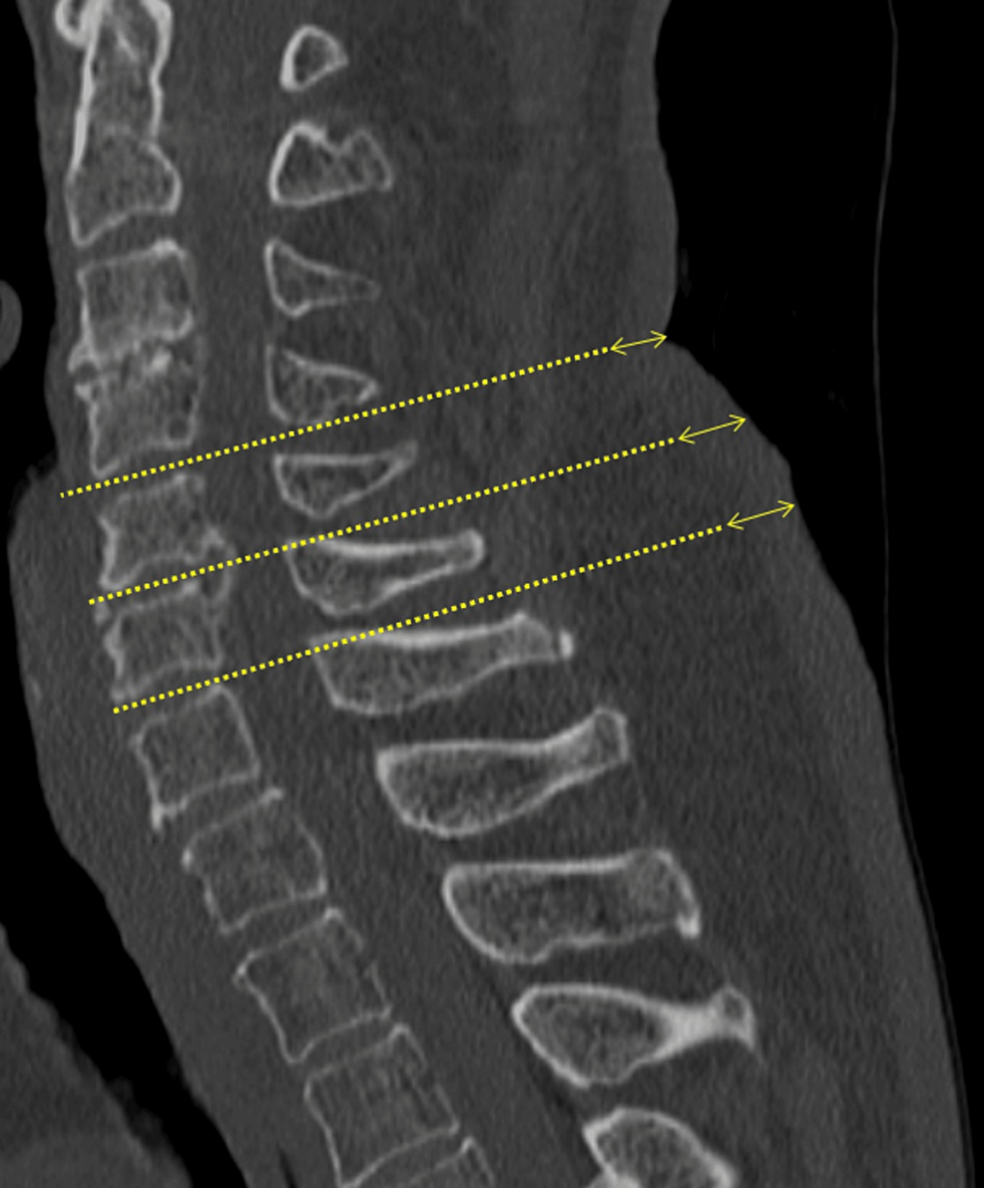
Measurement of cervical spine skin thickness using sagittal whole-spine CT. Dashed line: extension of the intervertebral disc. Bidirectional arrows: skin thickness. In this case, the skin thickness (average) of the cervical skin is 7.7 mm (7.3 mm at the C4-5 level, 8 mm at the C5-6 level, and 7.9 mm at the C6-7 level).

Analysis was performed using a 64-slice detector CT scanner (Canon Aquilion; Canon Medical System Co., Tochigi, Japan) with 1.0 mm thick axial slices in the bone window setting (window: 2000; level: 200). Skin thickness was categorized into two groups based on the median value using a cutoff of 4 mm. The average skin thickness at the cervical (C4-5, C5-6, C6-7 disc levels), thoracic (T7-8, T8-9, T9-10 disc levels), and lumbar (L2-3, L3-4, L4-5 disc levels) regions was determined from sagittal cross-sectional images using whole spine CT (Figures 1, 2).

Imaging

We investigated BMD, bone osteophyte formation, and bone proliferative diseases. BMD was evaluated by preoperative BMD testing (lumbar spine L1-L4 and total hip) using dual-energy X-ray absorptiometry, with measurements referenced to young adult mean values. In Japan, the criteria for osteoporosis diagnosis, formulated in 1996, were originally derived from the percentage of young adults with a mean areal BMD of the lumbar spine or femoral neck. Osteoporosis was defined as a fragility fracture in a young adult with a mean BMD of 70% or below [11]. The diagnosis of OPLL and evaluation of ossified OPLL lesions were made using CT scans of the cervical, thoracic, and lumbar spine. Radiographic and biochemical evaluations ruled out metabolic diseases associated with OPLL, including hypophosphatemic rickets, osteomalacia, and hyperparathyroidism. DISH was diagnosed based on the continuous ossification of the anterior spinal ligament involving four or more vertebrae, with degenerative disc disease being excluded [12]. The ossification index of all vertebrae was calculated by summing the vertebral bodies and intervertebral bridging osteophytes in the anterior and posterior regions of all vertebrae according to the method of Kawaguchi et al. [13,14]. The sum of the vertebral body and intervertebral bridging osteophytes of the anterior longitudinal and posterior longitudinal ligaments were defined as the OALL index and OPLL index, respectively.

Laboratory

Serum bone-related markers, such as tartrate-resistant acid phosphatase type 5b (TRACP5b), procollagen I N-propeptide (P1NP), 25-hydroxyvitamin D (25OHD), intact parathyroid hormone (PTH), calcium (Ca), phosphate (P), estimated glomerular filtration rate (eGFR), and periostin, were measured. Periostin is a secreted extracellular matrix protein expressed in collagen-rich fibrous connective tissue [15]. Its production is induced by fibroblasts in the skin and osteoblasts in the bone. Elevated periostin levels correlate with inflammatory skin diseases and OPLL [15,16].

Statistical analysis

The normality of data distribution was assessed using the Shapiro-Wilk test. Quantitative data with non-normal distributions were presented as medians (interquartile range) and compared using the Kruskal-Wallis test (for three groups).

To evaluate the association between skin thickness and imaging findings, univariate logistic regression analysis was performed to calculate crude odds ratio (OR), while multivariate logistic regression analysis was used for calculating adjusted OR after accounting for age (years, continuous), sex (0: male, 1: female), and body mass index (BMI; kg/m^2^, continuous). Skin thickness (0: <4 mm, 1: ≥4.0 mm) was considered the dependent variable, while images (0: absent, 1: present) were defined as the independent variable. The absence of images served as the reference category, with an OR of 1.00, and the ORs for the other categories were interpreted relative to this reference.

To assess the association between skin thickness, images, and laboratory test results, a simple regression analysis was performed using the crude partial regression coefficient (B). Multivariate regression analysis was performed using the adjusted B controlled for age (years, continuous), sex (0: male, 1: female), and BMI (kg/m^2^, continuous). Skin thickness (mm, continuous) was used as the dependent variable, while images or laboratory test results (continuous) were used as independent variables.

All analyses were performed using JMP® Pro 16 software (SAS Institute, Cary, NC, USA). As skin thickness was evaluated separately for the cervical, thoracic, and lumbar levels, the significance level was adjusted using the Bonferroni correction (0.05/3), with a p-value of 0.017 considered statistically significant.

## Results

Clinical characteristics of the cohort

Table 1 presents the clinical characteristics of the cohort. The mean age of the participants was 74.3 years, with 42.4% being women. Analysis revealed no significant differences in skin thickness across the three dorsal regions spanning the cervical, thoracic, and lumbar spine (median = 3.3 mm versus 3.5 mm versus 3.4 mm, Kruskal-Wallis test, statistic H = 2.058, p = 0.357).

**Table 1:** Demographics, image, and laboratory data of the study cohort. ^a^: Values are presented as mean ± standard deviation. ^b^: Values are presented as median (interquartile range). ^c^: Values are presented as number (percentage). BMD = bone mineral density; OPLL = ossification of the posterior longitudinal ligament; DISH = diffuse idiopathic skeletal hyperostosis; TRACP5b = tartrate-resistant acid phosphatase type 5b; P1NP = procollagen I N-propeptide; 25OHD = 25-hydroxyvitamin D; PTH = parathyroid hormone; eGFR = estimated glomerular filtration rate

Demographics	(n = 99)
Age^a^, years	74.3 ± 7.2
Female^c^, n (%)	42 (42.4)
Body mass index^b^, kg/m^2^	23.4 (21.2–27.0)
Image	
Cervical skin thickness^b^, mm	3.3 (2.6–4.2)
Thoracic skin thickness^b^, mm	3.5 (2.7–4.4)
Lumbar skin thickness^b^, mm	3.4 (2.6–3.9)
BMD lumbar spine^b^, %	101.0 (87.0–115.0)
BMD total hip^b^, %	85.0 (75.0–94.0)
Osteoporosis^c^, n (%)	25 (25.2)
DISH^c^, n (%)	32 (32.3)
OPLL^c^, n (%)	22 (22.2)
Laboratory	
TRACP5b^b^, mU/dL	409.0 (313.0–555.0)
P1NP^b^, μg/L	46.4 (33.8–67.4)
25OHD^b^, ng/mL	16.1 (10.7–20.8)
Intact PTH^b^, pg/mL	51.0 (37.0–68.0)
Ca^a^, mg/dL	9.1 ± 0.4
P^b^, mg/dL	3.4 (3.0–3.7)
eGFR^b^, mL/minute/1.73m^2^	65.8 (51.7–78.2)
Periostin^b^, ng/mL	35.0 (27.8–40.1)

The demographics, imaging, and laboratory parameters between cervical, thoracic, and lumbar skin thickness of ≥4.0 mm and <4.0 mm are summarized in Table 2.

**Table 2:** Demographics, image, and laboratory data between skin thickness of ≥4.0 mm and <4.0 mm. ^a^: Values are presented as mean ± standard deviation. ^b^: Values are presented as median (interquartile range). ^c^: Values are presented as number (percentage). BMD = bone mineral density; OPLL = ossification of the posterior longitudinal ligament; OALL = ossification of the anterior longitudinal ligament; DISH = diffuse idiopathic skeletal hyperostosis; TRACP5b = tartrate-resistant acid phosphatase type 5b; P1NP = procollagen I N-propeptide; 25OHD = 25-hydroxyvitamin D; PTH = parathyroid hormone; eGFR = estimated glomerular filtration rate

	Cervical skin thickness		Thoracic skin thickness		Lumbar skin thickness	
	≥4.0 mm (n = 28)	<4.0 mm (n = 71)	≥4.0 mm (n = 38)	<4.0 mm (n = 61)	≥4.0 mm (n = 24)	<4.0 mm (n = 75)
Demographics						
Age, years^a^	72.7 ± 7.6	75.0 ± 7.0	72.5 ± 7.2	75.4 ± 7.1	73.1 ± 8.6	74.7 ± 6.7
Female^c^, n (%)	7 (25.0)	35 (49.3)	10 (26.3)	32 (52.5)	11 (45.8)	31 (41.3)
Body mass index^b^, kg/m^2^	25.2 (23.3–28.5)	22.3 (20.1–26.2)	24.8 (21.9–28.6)	22.7 (20.5–25.8)	27.3 (24.3–29.4)	22.7 (20.9–25.2)
Image						
BMD lumbar spine^b^, %	110.0 (99.0–137.5)	96.0 (85.0–111.0)	104.5 (89.5–115.5)	100.0 (85.0–115.0)	110.0 (88.3–125.3)	99.0 (86.0–112.0)
BMD total hip^b^, %	94.0 (83.5–102.0)	81.0 (71.0–93.0)	90.0 (79.8–98.3)	81.0 (71.0–93.5)	91.0 (80.0–99.0)	82.0 (71.0–94.0)
Osteoporosis^c^, n (%)	3 (10.7)	22 (31.0)	5 (13.2)	20 (32.8)	4 (16.7)	21 (28.0)
OALL index^b^	0 (0–0)	0 (0–0)	0 (0–0)	0 (0–0)	0 (0–0)	0 (0–0)
OPLL index^b^	0 (0–1)	0 (0–0)	0 (0–1)	0 (0–0)	0 (0–2)	0 (0–0)
DISH, n (%)	16 (57.1)	16 (22.5)	15 (39.5)	17 (27.9)	8 (33.3)	24 (32.0)
OPLL, n (%)	9 (32.1)	13 (18.3)	11 (29.0)	11 (18.0)	11 (45.8)	11 (14.7)
Laboratory						
TRACP5b^b^, mU/dL	354.0 (285.3–539.0)	421.0 (326.0–559.0)	377.0 (303.3–551.3)	421.0 (331.5–557.0)	354.0 (277.0–434.5)	428.0 (326.0–573.0)
P1NP^b^, μg/L	43.5 (34.1–55.4)	54.1 (33.8–80.2)	41.2 (31.4–57.3)	55.3 (36.8–77.3)	43.5 (31.6–59.7)	50.0 (34.2–77.5)
25OHD^b^, ng/mL	17.1 (13.4–20.9)	16.0 (9.9–20.8)	16.6 (13.3–20.3)	16.0 (9.8–21.2)	14.3 (8.1–18.8)	16.8 (10.7–21.6)
Intact PTH^b^, pg/mL	42.5 (33.3–60.5)	52.0 (39.0–71.0)	51.5 (38.5–63.3)	50.0 (36.0–72.0)	55.0 (43.0–66.8)	49.0 (36.0–70.0)
Ca^a^, mg/dL	9.1 ± 0.4	9.1 ± 0.4	9.1 ± 0.4	9.1 ± 0.5	9.2 ± 0.4	9.1 ± 0.4
P^b^, mg/dL	3.3 (2.9–3.5)	3.4 (3.0–3.9)	3.3 (2.8–3.5)	3.5 (3.1–3.9)	3.3 (2.7–4.0)	3.4 (3.0–3.7)
eGFR^b^, mL/minute/1.73m^2^	65.5 (53.6–72.9)	66.9 (48.9–78.4)	65.7 (53.5–82.2)	66.9 (49.8–76.4)	65.5 (50.6–75.2)	66.9 (51.7–78.7)
Periostin^b^, ng/mL	30.2 (24.4–41.5)	35.6 (28.3–40.1)	31.8 (27.7–40.3)	36.0 (28.0–40.2)	35.4 (26.5–39.3)	34.8 (27.9–44.6)

Regression analyses

The relationships between skin thickness, imaging findings, and laboratory test results are presented in Table 3 and Table 4. In the univariate logistic regression analyses, cervical, thoracic, and lumbar skin thicknesses were found to be associated with DISH, BMD total hip, OPLL index, and P; osteoporosis, BMD total hip, OPLL index, and P; and OPLL, BMD total hip, and OPLL index, respectively. Upon adjusting for age, sex, and BMI, cervical, thoracic, and lumbar skin thicknesses were found to be associated with DISH, whereas no significant association was observed with BMD or osteoporosis; OPLL index but not P; and OPLL and OPLL index, respectively.

**Table 3:** Results of univariate and multivariate logistic regression analyses showing the relationship between the skin thickness and images. ^a^: Adjusted for age (years, continuous), sex (0: male, 1: female), and body mass index (kg/m^2^, continuous). ^b^: The Bonferroni method defined the significance level as 0.05/3 ≈ 0.017. OR = odds ratio; CI = confidence interval; OPLL = ossification of the posterior longitudinal ligament; DISH = diffuse idiopathic skeletal hyperostosis

Dependent variable	Independent variable	Crude OR (95% CI)	P-value	Adjusted OR^a^ (95% CI)	P-value^b^
Cervical skin thickness ≥4.0 mm	Osteoporosis, absent	1.00 (Reference)		1.00 (Reference)	
	Osteoporosis, present	0.27 (0.07 to 0.98)	0.047	0.46 (0.11 to 1.83)	0.268
	DISH, absent	1.00 (Reference)		1.00 (Reference)	
	DISH, present	4.58 (1.80 to 11.6)	0.001	4.44 (1.58 to 12.5)	0.005
	OPLL, absent	1.00 (Reference)		1.00 (Reference)	
	OPLL, present	2.11 (0.78 to 5.72)	0.141	1.44 (0.50 to 4.19)	0.501
Thoracic skin thickness ≥4.0 mm	Osteoporosis, absent	1.00 (Reference)		1.00 (Reference)	
	Osteoporosis, present	0.31 (0.11 to 0.92)	0.034	0.48 (0.15 to 1.57)	0.227
	DISH, absent	1.00 (Reference)		1.00 (Reference)	
	DISH, present	1.69 (0.72 to 3.98)	0.232	1.48 (0.57 to 3.88)	0.418
	OPLL, absent	1.00 (Reference)		1.00 (Reference)	
	OPLL, present	1.85 (0.71 to 4.83)	0.207	1.24 (0.44 to 3.48)	0.683
Lumbar skin thickness ≥4.0 mm	Osteoporosis, absent	1.00 (Reference)		1.00 (Reference)	
	Osteoporosis, present	0.51 (0.16 to 1.68)	0.272	0.65 (0.18 to 2.36)	0.513
	DISH, absent	1.00 (Reference)		1.00 (Reference)	
	DISH, present	1.06 (0.40 to 2.82)	0.903	0.93 (0.32 to 2.71)	0.898
	OPLL, absent	1.00 (Reference)		1.00 (Reference)	
	OPLL, present	4.92 (1.76 to 13.74)	0.002	4.78 (1.58 to 14.41)	0.006

**Table 4:** Results of univariate and multivariate regression analyses showing the relationship between the skin thickness and images and laboratories. ^a^: Adjusted for age (years, continuous), sex (0: male, 1: female), and body mass index (kg/m^2^, continuous). ^b^: The Bonferroni method defined the significance level as 0.05/3 ≈ 0.017. B = regression coefficient; CI = confidence interval; TRACP5b = tartrate-resistant acid phosphatase type 5b; P1NP = procollagen I N-propeptide; 25OHD = 25-hydroxyvitamin D; PTH = parathyroid hormone; eGFR = estimated glomerular filtration rate

Dependent variable	Independent variable	Crude B (95% CI)	P-value	Adjusted B^a^ (95% CI)	P-value^b^
Cervical skin thickness, mm	BMD lumbar spine, %	0.01 (0 to 0.03)	0.046	0 (–0.01 to 0.01)	0.995
	BMD total hip, %	0.03 (0.01 to 0.05)	0.004	0.01 (–0.01 to 0.03)	0.404
	OALL index	–0.34 (–1.59 to 0.90)	0.583	– 0.43 (–1.59 to 0.73)	0.462
	OPLL index	0.16 (0.03 to 0.29)	0.019	0.10 (–0.04 to 0.23)	0.154
	TRACP5b, mU/dL	0 (0 to 0)	0.252	0 (0 to 0)	0.243
	P1NP, μg/L	0 (0 to 0)	0.195	0 (0 to 0)	0.136
	25OHD, ng/mL	0.02 (–0.02 to 0.06)	0.348	0 (–0.03 to 0.05)	0.775
	Intact PTH, pg/mL	–0.01 (–0.02 to 0)	0.074	–0.01 (–0.02 to 0)	0.070
	Ca, mg/dL	0.30 (–0.39 to 1.00)	0.395	0.50 (–0.15 to 1.16)	0.132
	P, mg/dL	–0.62 (–1.10 to –0.15)	0.010	–0.35 (–0.83 to 0.14)	0.159
	eGFR, mL/minute/1.73m^2^	0 (–0.02 to 0.01)	0.710	0 (–0.01 to 0.01)	0.741
	Periostin, ng/mL	0 (–0.02 to 0.04)	0.633	0 (–0.02 to 0.03)	0.707
Thoracic skin thickness, mm	BMD lumbar spine, %	0.01 (0 to 0.02)	0.121	0 (–0.02 to 0.01)	0.464
	BMD total hip, %	0.02 (0 to 0.04)	0.006	0.01 (–0.01 to 0.02)	0.505
	OALL index	–0.47 (–1.51 to 0.58)	0.377	–0.65 (–1.59 to 0.30)	0.178
	OPLL index	0.27 (0.17 to 0.37)	<0.001	0.23 (0.14 to 0.33)	<0.001
	TRACP5b, mU/dL	0 (0 to 0)	0.963	0 (0 to 0)	0.883
	P1NP, μg/L	0 (0 to 0)	0.245	0 (0 to 0)	0.245
	25OHD, ng/mL	–0.02 (–0.05 to 0.02)	0.414	–0.03 (–0.06 to 0)	0.072
	Intact PTH, pg/mL	0 (–0.01 to 0)	0.527	0 (–0.01 to 0)	0.703
	Ca, mg/dL	–0.07 (–0.66 to 0.52)	0.811	0.11 (–0.43 to 0.66)	0.690
	P, mg/dL	–0.77 (–1.15 to –0.38)	<0.001	–0.52 (–0.91 to –0.13)	0.009
	eGFR, mL/minute/1.73m^2^	0 (0 to 0.02)	0.365	0 (0 to 0.01)	0.366
	Periostin, ng/mL	0 (–0.02 to 0.02)	0.992	0 (–0.02 to 0.02)	0.811
Lumbar skin thickness, mm	BMD lumbar spine, %	0.01 (0 to 0.02)	0.085	0 (0 to 0.01)	0.600
	BMD total hip, %	0.02 (0.01 to 0.03)	0.001	0.02 (0 to 0.03)	0.047
	OALL index	–0.51 (–1.39 to 0.37)	0.251	–0.49 (–1.36 to 0.38)	0.264
	OPLL index	0.23 (0.15 to 0.32)	<0.001	0.22 (0.13 to 0.31)	<0.001
	TRACP5b, mU/dL	0 (0 to 0)	0.084	0 (0 to 0)	0.110
	P1NP, μg/L	0 (0 to 0)	0.237	0 (0 to 0)	0.259
	25OHD, ng/mL	–0.02 (–0.05 to 0.01)	0.174	–0.03 (–0.06 to 0)	0.056
	Intact PTH, pg/mL	0 (0 to 0.01)	0.394	0 (0 to 0.01)	0.402
	Ca, mg/dL	–0.02 (–0.52 to 0.48)	0.934	0.05 (–0.45 to 0.55)	0.847
	P, mg/dL	–0.26 (–0.61 to 0.09)	0.139	–0.14 (–0.51 to 0.22)	0.432
	eGFR, mL/minute/1.73m^2^	0 (0 to 0.01)	0.839	0 (0 to 0.01)	0.626
	Periostin, ng/mL	–0.01 (–0.03 to 0.001)	0.199	–0.01 (–0.03 to 0.01)	0.177

## Discussion

The main outcomes of this study were the skin-bone relationships: DISH with cervical skin thickness, OPLL index with thoracic skin thickness, and OPLL index. The secondary outcomes included (1) no correlation between skin thickness and BMD; (2) no correlation between bone-related markers such as TRACP5b, P1NP, 25OHD, intact PTH, Ca, eGFR, P, and periostin; and (3) no significant difference in skin thickness over the cervical, thoracic, and lumbar spine.

The skin and bone may share a common pathology because they share a common component, collagen [7]. Collagen in the skin and bone is also affected by aging, hormones, and drugs, each with a common matrix such as estrogen [17,18], growth hormone [19,20], and corticosteroids [21] which can have positive or negative effects. Risk factors for osteoporosis, such as steroid use, menopause, and undernutrition (BMI <20 kg/m^2^) have been reported to decrease skin thickness [9]. Therefore, the pathological modification of collagen can be inferred as a common predisposition to skin thinning and osteoporosis. In a systematic review including 14 articles on the association between skin thickness and osteoporosis by Aurégan et al. [9], seven of the eight studies that examined skin thickness and BMD showed significant correlations (R ranging between 0.19 and 0.486). However, the correlation coefficients were low, which may have been due to the low accuracy of BMD measurements before 1990 and the lack of uniformity in skin thickness measurement sites, mainly because skin thinness and osteoporosis may both be multifactorial [9]. We adjusted for age, sex, and BMI to exclude potential confounders.

Therefore, to exclude potential confounders, the study was adjusted for age, gender and BMI. To our knowledge, our study is the first clinical study to find an association between skin thickness and osteoproliferative disorders (OPLL, DISH). BMD reflects the density of the vertebral trabecular bone; bone proliferative disorders (OPLL and DISH) reflect ligament ossification. Therefore, skin thickness may reflect ligament ossification better compared with bone density. Pathological investigations have reported thicker skin in patients with OPLL and proliferation of the extracellular matrix bound to type I collagen fibers in the dermal layer [22]. Therefore, ligament ossification and bony spurs may correlate more strongly with skin collagen proliferation.

OPLL and DISH have been reported to be associated with low-level chronic inflammation throughout the body [23,24]. Moreover, metabolic syndrome, diabetes mellitus, and obesity, which are highly comorbid with OPLL and DISH, also have been revealed to be associated with low-level chronic inflammation throughout the body [25-28]. Skin inflammation may result in increased vascular permeability, edema, and swelling. Due to the infiltration of inflammatory cells within the dermis and hyperplasia of the epidermis, skin thickness may increase [29]. In bone, palmoplantar pustulosis, a type of systemic inflammatory skin disease, has been characterized by an increase in inflammatory cytokines, which can induce not only arthritis but also increased osteoproliferation. Common inflammatory pathways may underlie osteoproliferative disease (OPLL and DISH) and increased skin thickness or inflammatory skin disease. Taken together, patients with thicker skin were suspected to have more vertebral osteophytes, OPLL, and DISH. Therefore, skin thickness may be a biomarker for spinal osteoproliferative disorders, such as OPLL and DISH.

Second, we focused on TRACP5b, P1NP, 25OHD, intact PTH, Ca, P, eGFR, and periostin as bone-related markers and investigated their association with skin thickness. To our knowledge, this is the initial study to investigate the relationship between skin thickness and bone-related markers. PINP is cleaved from type I pro-collagen. As approximately 70% of type I collagen is present in bone tissue, PINP is used as an osteogenic marker [30]. TRACP5b is an enzyme produced by osteoclasts and used as a bone resorption marker [30]. PINP and TRACP5b are used to determine the bone metabolic turnover, with high and low values indicating high and low turnover, respectively. As bone-related markers such as PINP, TRACP5b, 25OHD, intact PTH, Ca, P, and eGFR affect or reflect bone formation and resorption, they are considered direct and indirect indicators of bone turnover. As there was no correlation between bone-related markers without P and skin thickness, bone turnover may not be correlated with skin thickness.

Periostin is predominantly expressed in connective tissues that experience mechanical loading, such as the skin, bone, periodontal ligament, and heart valves. During pathogenesis, periostin plays a variety of roles, including skin wound healing, osteogenesis, angiogenesis, tissue remodeling, fibrosis, inflammation, and cancer formation [15]. As fibroblasts, which produce collagen in the skin, secrete periostin, we hypothesized that there might be a correlation between serum periostin and skin thickness. No correlation was found between these two variables. Because periostin is ubiquitously expressed for many causes, its association with skin thickness may be difficult to detect.

This study has several limitations. First, there are racial differences in skin thickness, with the skin of Asian people tending to be thicker than that of Caucasians [31]. As this study included an all-Japanese population, the results may not apply to other races. Second, although we adjusted for key confounders, the results may still be biased owing to the paucity of cases and retrospective nature of this study. Third, the measurement site was the dorsal to the spine. In general, the skin on the trunk tends to be thicker than that on the extremities [32], suggesting that the dorsal spine may be more likely, compared with the extremities, to show significant differences in skin thickness studies. However, unlike the spine, data of the extremities could easily be used to measure skin thickness by ultrasound examination of the extremities without removing clothing or considering embarrassment. Data of the extremities would be warranted for further investigation.

## Conclusions

The impact of OPLL and DISH on quality of life and socioeconomic factors would increase as the number of patients with OPLL and DISH increases with the aging of society.

Dorsal skin thickness measurements over the cervical, thoracic, and lumbar spines may be used to screen for abnormal bone metabolism, including osteoproliferative disorders such as OPLL and DISH. Further prospective, large, cohort studies are required to confirm skin thickness as a diagnostic biomarker for proliferative bone diseases. A thorough elucidation of the relationship between skin and bone may lead to a deeper insight into the pathophysiology of the disease.

The study of bone-skin interactions is a new frontier and may meet the unmet clinical needs for an ever-increasing number of people with bone metabolic diseases.

## References

[1] Hsu SC, Feng SH, Pan SL (2023). Risk of developing age-related macular degeneration in patients with osteoporosis: a population-based, longitudinal follow-up study. Osteoporos Int.

[2] Mader R, Verlaan JJ, Eshed I (2017). Diffuse idiopathic skeletal hyperostosis (DISH): where we are now and where to go next. RMD Open.

[3] Kawaguchi Y, Imagama S, Iwasaki M (2021). Japanese Orthopaedic Association (JOA) clinical practice guidelines on the management of ossification of the spinal ligament, 2019. J Orthop Sci.

[4] Ross FP, Christiano AM (2006). Nothing but skin and bone. J Clin Invest.

[5] Choi KY, Ajiteru O, Hong H (2023). A digital light processing 3D-printed artificial skin model and full-thickness wound models using silk fibroin bioink. Acta Biomater.

[6] Kitagawa T, Matsuda K, Inui S (2009). Keratinocyte growth inhibition through the modification of Wnt signaling by androgen in balding dermal papilla cells. J Clin Endocrinol Metab.

[7] Morimoto T, Hirata H, Sugita K (2024). A view on the skin-bone axis: unraveling similarities and potential of crosstalk. Front Med (Lausanne).

[8] Engman MF (1919). The skin: a mirror to the system. JAMA.

[9] Aurégan JC, Bosser C, Bensidhoum M, Bégué T, Hoc T (2018). Correlation between skin and bone parameters in women with postmenopausal osteoporosis: a systematic review. EFORT Open Rev.

[10] Leal JM, de Souza GH, Marsillac PF, Gripp AC (2021). Skin manifestations associated with systemic diseases - part II. An Bras Dermatol.

[11] Soen S, Fukunaga M, Sugimoto T (2013). Diagnostic criteria for primary osteoporosis: year 2012 revision. J Bone Miner Metab.

[12] Resnick D, Niwayama G (1976). Radiographic and pathologic features of spinal involvement in diffuse idiopathic skeletal hyperostosis (DISH). Radiology.

[13] Hirai T, Yoshii T, Nagoshi N (2018). Distribution of ossified spinal lesions in patients with severe ossification of the posterior longitudinal ligament and prediction of ossification at each segment based on the cervical OP index classification: a multicenter study (JOSL CT study). BMC Musculoskelet Disord.

[14] Kawaguchi Y, Nakano M, Yasuda T (2016). Characteristics of ossification of the spinal ligament; incidence of ossification of the ligamentum flavum in patients with cervical ossification of the posterior longitudinal ligament - analysis of the whole spine using multidetector CT. J Orthop Sci.

[15] Takeshita S, Kikuno R, Tezuka K, Amann E (1993). Osteoblast-specific factor 2: cloning of a putative bone adhesion protein with homology with the insect protein fasciclin I. Biochem J.

[16] Kawaguchi Y, Kitajima I, Yasuda T (2022). Serum periostin level reflects progression of ossification of the posterior longitudinal ligament. JB JS Open Access.

[17] Affinito P, Palomba S, Sorrentino C, Di Carlo C, Bifulco G, Arienzo MP, Nappi C (1999). Effects of postmenopausal hypoestrogenism on skin collagen. Maturitas.

[18] Sumino H, Ichikawa S, Abe M (2004). Effects of aging and postmenopausal hypoestrogenism on skin elasticity and bone mineral density in Japanese women. Endocr J.

[19] Shuster S (2020). Osteoporosis, like skin ageing, is caused by collagen loss which is reversible. J R Soc Med.

[20] Black MM, Shuster S, Bottoms E (1972). Skin collagen and thickness in acromegaly and hypopituitarism. Clin Endocrinol (Oxf).

[21] Oikarinen A, Autio P (1991). New aspects of the mechanism of corticosteroid-induced dermal atrophy. Clin Exp Dermatol.

[22] Imamura T, Sakou T, Matsunaga S, Taketomi E, Ishido Y, Yoshida H (1995). Histochemical and immunohistochemical study on the skin of patients with ossification of the posterior longitudinal ligament in the cervical spine. In Vivo.

[23] Sarzi-Puttini P, Atzeni F (2004). New developments in our understanding of DISH (diffuse idiopathic skeletal hyperostosis). Curr Opin Rheumatol.

[24] Kawaguchi Y, Nakano M, Yasuda T (2017). Serum biomarkers in patients with ossification of the posterior longitudinal ligament (OPLL): inflammation in OPLL. PLoS One.

[25] Argano C, Mirarchi L, Amodeo S, Orlando V, Torres A, Corrao S (2023). The role of vitamin D and its molecular bases in insulin resistance, diabetes, metabolic syndrome, and cardiovascular disease: state of the art. Int J Mol Sci.

[26] Zatterale F, Longo M, Naderi J, Raciti GA, Desiderio A, Miele C, Beguinot F (2019). Chronic adipose tissue inflammation linking obesity to insulin resistance and type 2 diabetes. Front Physiol.

[27] Hao X, Shang X, Liu J, Chi R, Zhang J, Xu T (2021). The gut microbiota in osteoarthritis: where do we stand and what can we do?. Arthritis Res Ther.

[28] Morimoto T, Kobayashi T, Kakiuchi T (2023). Gut-spine axis: a possible correlation between gut microbiota and spinal degenerative diseases. Front Microbiol.

[29] Kalyan Kumar G, Dhamotharan R, Kulkarni NM, Mahat MY, Gunasekaran J, Ashfaque M (2011). Embelin reduces cutaneous TNF-α level and ameliorates skin edema in acute and chronic model of skin inflammation in mice. Eur J Pharmacol.

[30] Nakatoh S (2017). Bone turnover rate and bone formation/resorption balance during the early stage after switching from a bone resorption inhibitor to denosumab are predictive factors of bone mineral density change. Osteoporos Sarcopenia.

[31] Oltulu P, Ince B, Kokbudak N, Findik S, Kilinc F (2018). Measurement of epidermis, dermis, and total skin thicknesses from six different body regions with a new ethical histometric technique. Turk J Plast Surg.

[32] Varila E, Sievänen H, Vuori I, Oksanen H, Punnonen R (1995). Limited value of ultrasound measured skin thickness in predicting bone mineral density in peri- and postmenopausal women. Maturitas.

